# Deciphering the ecology of the threatened microendemic species *Euphorbia margalidiana*

**DOI:** 10.3389/fpls.2023.1155896

**Published:** 2023-06-26

**Authors:** Leonardo Llorens, Lucas Cortés, Herminio Boira

**Affiliations:** ^1^ Interdisciplinary Ecology Group, Department of Biology, University of the Balearic Islands (UIB), Palma de Mallorca, Balearic Islands, Spain; ^2^ Independent Researcher, Palma de Mallorca, Spain; ^3^ Institute of Mediterranean Agroforestry (IAM), Polytechnic University of Valencia (UPV), Valencia, Spain

**Keywords:** seed germination, seed dispersal, drought tolerance, flower scent, pollinators, conservation

## Abstract

Small islands play a critical role in the study of plant ecology and evolution. Here, we reveal the ecology of *Euphorbia margalidiana*, an endemic plant that thrives in a micro-island environment in the Western Mediterranean region. Through a detailed characterization of the habitat, including plant communities, microclimate, soil properties, and germination assays, we examine the effects of biotic and abiotic factors on the distribution of this endangered species. We also analyze its pollination biology, evaluate the success of vegetative propagation, and discuss its potential use in conservation strategies. Our results show that *E. margalidiana* is a characteristic species of the shrub ornitocoprophilous insular vegetation of the Western Mediterranean. The seeds have a very low dispersion capacity outside the islet and that seed-derived plants have higher survival rates under drought conditions than those vegetatively propagated. The main volatile compound emitted from the pseudanthia is phenol which attracts the plants’ main and almost exclusive pollinators in the islet, flies. Our results confirm the relictual status of *E. margalidiana* and highlight the importance of key adaptive traits that enable the survival of this species in the harsh micro-island environment of Ses Margalides.

## 1 Introduction

While islands make up less than 7% of the Earth’s surface, their importance in the conservation of the Earth’s biodiversity is very high, as they are home to approximately 20% of the planet’s species, including numerous endemics and threatened species (Fernández-Palacios et al., 2021).

Despite the image that many idealizations offer of islands as easy and ideal environments for life, the climatic, edaphic, and geomorphological characteristics of many of them, especially those of small size, determine that the reality is less idyllic. However, these environmental conditions can create ecological opportunities for some colonizers with peculiar traits that allow them to develop successfully. The habitats in these islands are highly vulnerable due to their high dependence on specific ecological agents. Therefore, small changes in soil, climate, or fauna, such as bird nesting sites (Wetzel et al., 2013), can have dramatic consequences for the terrestrial species that depend on them (habitat loss).

The use of islands as model systems is a recognized phenomenon around the world, and MacArthur and Wilson (1967) model is the foremost example. Recent island-based models have incorporated speciation (see Rosindell and Phillimore, 2011; Weigelt et al., 2013). However, as proposed by Warren et al. (2015), these models should incorporate the diverse modes of speciation and conservation, in geographic-ecological speciation. In this model, geographic isolation is the component initiating genetic divergence and ecological speciation as a result of divergent selection.

The ecological and biogeographical interest of the small island biota is due to the relatively simple and well-defined characteristics of their environments which are largely determined by factors such as nesting seabird colonies (Gillham, 1961; Ghazanfar et al., 2001) or grazing (Bergmeier and Dimopoulos, 2003). Additionally, other abiotic factors include the absence of a well-developed soil layer, and severe drought or extreme daily temperature ranges (Vogiatzakis and Griffiths, 2008). As a result, interspecific competition between plants is low on the islets as species need to adapt to certain ecological niches. It has been suggested that the major factors responsible for the low species richness of small islands are the sporadic arrival of water and/or bird-dispersed seeds and then finding a suitable substrate for the successful establishment of plants. In many of these Mediterranean islets, the habitat conditions are determined by the influence of the sea which creates conditions similar to those found on the cliff sides. Coastal rocky habitats, which are resource-poor (Larson et al., 2000), experience low species diversity due to additional stress factors like salinity and seabird presence. Resource scarcity is caused by the limited availability of nutrients (excluding those from seabirds) and space for root growth and biotic recruitment. Despite these harsh conditions, some species flourish in these habitats, which may provide some benefits such as increased protection from climatic extremes, herbivores, and human disturbance.

The islets flora is a combination of variable populations of ephemeral herbaceous and long-lived shrubs, with rates of recruitment and mortality low or very low, which make up permanent plant communities (Eriksson and Ehrlén, 1992; García, 2003). Rechinger and Rechinger-Moser (1951) propose to use the concept of “islet specialist” to highlight certain taxa that only (or mostly) develop in small islands of low altitude, preferably located in exposed and isolated places. Rapid micro-speciation processes occur in these very small territories, which, combined with special biotic assemblages and interactions between a few species, are conducive to the islet specialists (Höner and Greuter, 1988). These species are highly specialized and well adapted to the ecological conditions of the islands, where they can be relatively abundant. However, in general, their limited area of distribution and the reduced possibility of developing new stable populations determines a high risk of extinction (Snogerup and Snogerup, 2004; Georghiou and Delipetrou, 2010). This causes many of them to have been included in lists of endangered species.

*Euphorbia margalidiana* Kühbier & Lewejohann constitutes an important example of an islet specialist. It is an endemic species, exclusive to the islet of Ses Margalides (Ibiza, Balearic Islands), with a population consisting of about 1000 plants (Conesa et al., 2005) and is integrated into the bush present in the limestone clifftops crevices. This species is an interesting model of plant speciation in a recently isolated island habitat. From a biological and ecological perspective, our study aims to: 1) determine the microclimatic and edaphic conditions of the native habitat of *E. margalidiana*; 2) compare the vegetation where it thrives to that of other islets in the Western Mediterranean region; 3) examine the unique traits (morphology, pollination, germination and dispersal) that contribute to the species’ adaptation and location on the islet; 4) gather experimental data for conservation, focusing on seedling survival under xeric conditions like those in their natural habitat.

## 2 Materials and methods

### 2.1 The site

**Figure 1:**
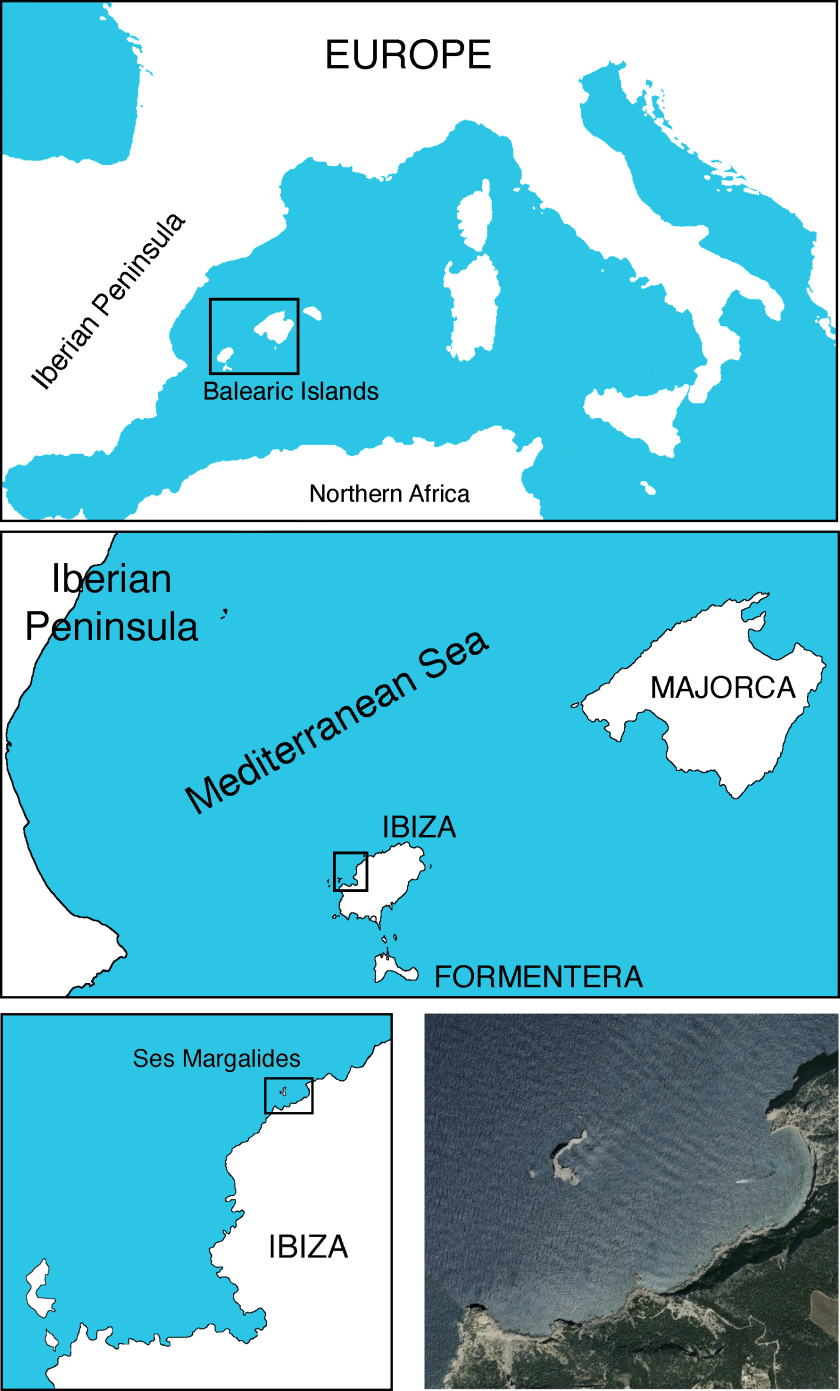
Location of Ses Margalides islets. Photos taken by the authors.

**Figure 2:**
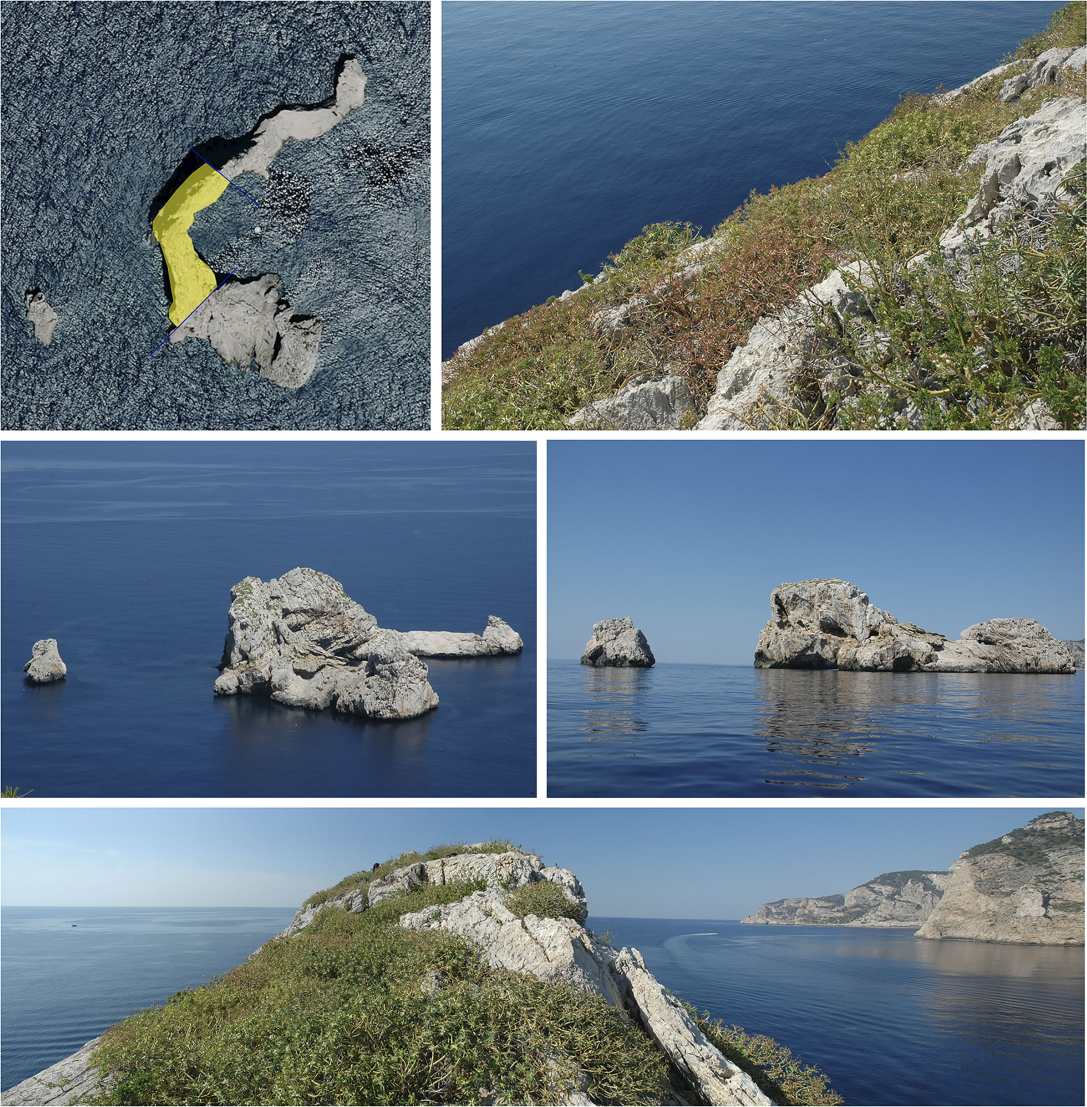
Morphology of Ses Margalides and distribution *E. margalidiana* vegetation in the summit area of Na Foradada. Photos taken by the authors.

Ses Margalides consist of two calcareous islets, Es Picatxó and Na Foradada, located approximately 500 meters from the northwest coast of Ibiza (39°02’57’’N, 1°18’54’’E) in the Western Mediterranean (**Figure 1**). Na Foradada, the larger of the two, spans 1.3 hectares and reaches a peak of 44 meters whereas the smaller one has no vegetation on it (for the purpose of this text, Ses Margalides refers to the larger island). Its notable features include steep walls and crevices, and the summit is home to the endemic *E. margalidiana* species (**Figure 2**). Vegetation is determined by saline mist, strong winds, nesting seabird colonies (marine gulls), and by the absence of grazing (no rabbits or goats exist in the islet). The main species of its flora are *Allium commutatum* Guss*.*, *E. margalidiana, Limonium ebusitanum* (Font Quer) Font Quer, *Malva arborea* (L.) Webb & Berthel.*,* and being less abundant: *Arthrocnemum macrostachyum* (Moric.) K. Koch, *Asparagus horridus* L*., Chenopodiastrum murale* (L.) S. Fuentes et al., *Crithmum maritimum* L., *Diplotaxis ibicensis* (Pau) Gómez-Campo, *Parietaria judaica* L., *Senecio gallicus* Vill. and *Sonchus tenerrimus* L.

Despite the high ecological significance of the islet and the presence of endangered species, the site has yet to be characterized in terms of its climatic or soil conditions. Therefore we measured temperature, pluviometry, relative humidity, and soil temperature. The thermo-pluviometric parameters obtained on the islet of Ses Margalides and those of the Sant Antoni station (Ibiza) are summarized in **Table 1**. All temperature values for the islet are higher than those for the coast of the main island, whereas the precipitation values are about 21.1% lower on the islet. Consequently, the islet is warmer and drier than the neighboring coast of Ibiza. According to Rivas Martínez and Rivas-Sáenz, (1996–2017), Margalides islet summit has a Mediterranean xeric-oceanic, upper inframediterranean upper semiarid microclimate (It= 463-473), and Sant Antoni coast has a Mediterranean xeric-oceanic, upper thermomediterranean, upper semiarid bioclimate (It=401).

**Table 1:** Values of climatic measures in the summit of Margalides islet and station of Sant Antoni de Portmany (Ibiza).

	Margalides (summit)		Sant Antoni de Portmany (Ibiza)
	Soil -5 cm	Soil +50 cm	Soil +200 cm
T (°C)	20.6	20.1	18.3
TMabs (°C)	36.1	42.6	34.8
Tmabs (°C)	5.7	5.1	0.1
TMjan (°C)	15.8	16.5	14.8
Tmjan (°C)	10.9	9.7	7.0
Pp (mm)	310.0		384.6
It	473	463	401

In Margalides: values in soil (-5 cm) and aerial (+50 cm). T: average annual temperature; TMabs: absolute maximum temperature; Tmabs: absolute minimum temperature; TMjan: average maximum January temperature; Tmjan: average minimum January temperature; Pp: precipitation (mm); It: Rivas-Martínez thermicity index.

The soil where *E. margalidiana* grows is largely composed of organic matter (%O.M. 39.7) due to the accumulation of bird droppings, dead vegetation, and other organic material, and has a sandy-loam texture with a neutral pH of 7.3. As a consequence, soils have a high Nitrogen content (%N: 0.56). Total CaCO_3_ has moderate levels (13.8%) with low quantities of active CaCO_3_ (0.7%). The soil is moderately saline with a conductivity of 4.1 mS/cm and a sodium adsorption ratio of 4.7.

### 2.2 Plant species

**Figure 3:**
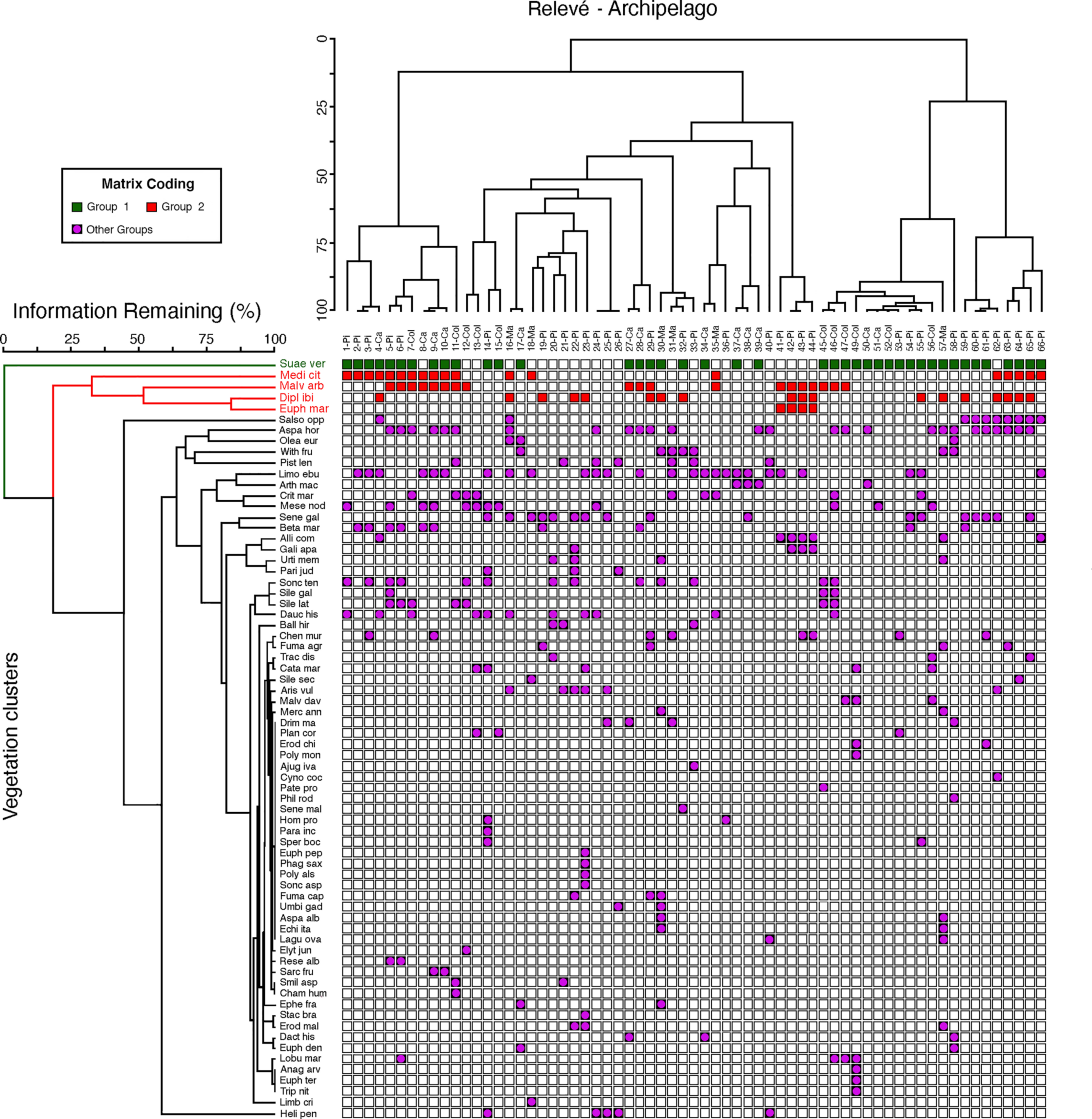
Main vegetation groups in islets from the Columbrets, Cabrera, Pityusic, and Majorca archipelagos in the Western Mediterranean Sea. Ses Margalides inventories (41-44). Green squares: S*uaeda vera* chamaephytic vegetation; red squares: nitrophilous chamaephytic vegetation (Medicagini citrinae-Lavaterion arboreae); and violet squares: less defined vegetation.

The Margalides spurge is a subsucculent dwarf-shrub, ramified, up to 100 cm in height. Stem erect or ascendent, smooth, glabrous, with a caudex of hypocotilar origin. Inflorescence (pleochasium) with five branches, three to five of them being furcated, often two or three of them being bifurcated. Cyathium (pseudanthium) 3-4 mm; nectary non-appendiculate, transverse elliptic, entire, yellowish. Fruit 7-9 X 7-10 mm, verrucose. Seeds 3,5-5 x 2,5-3 mm, dark grey or black (**Figure 3**). Taxonomically, *E. margalidiana* is included in the *E. squamigera* complex (subsect. *Galarrhaei* (Boiss.) Pax) (Vicens et al., 1997) together with seven to twelve other species, among which are E*. bivonae* Steud. and *E. papillaris* (Boiss.) Raffaelli & Ricceri, from Sicily. It is particularly close to *E. squamigera* Loisel., a species with the same chromosomal number (2n = 26) and similar karyotype (Simoín and Vicens, 1999; Barres et al., 2011).

### 2.3 Microclimatic data

Microclimatic data was obtained between 2011 and 2013 from a rocky crevice on the islet clifftop. Pluviometry, temperature, and relative humidity (RH) were measured at 40 cm above the soil level where *E. margalidiana* grows. For this purpose, an automatic pluviometer (RainGauge with Data logger Madgetech) and a Decagon EM50 data logger with Temp/RH and ECH2O TE sensors were used, respectively. The islet climatic data were compared to that of the nearby coast of Ibiza (Sant Antoni de Portmany station, 38°59’10 “N, 01°18’00” E), which is about 7 km away (http://infomet.am.ub.es/clima/eivissa/). The sensor for soil temperature and RH measurements was placed at 5 cm of depth in the soil.

### 2.4 Soil analysis

Soil samples were obtained from three crevices of the summit of an islet, colonized by *E. margalidiana*. The parameters of texture, pH, carbonates, active limestone, organic matter, conductivity, salt, cations (Ca2+, Mg2+, and Na+), and SAR (sodium absorption ratio) were determined. Soil texture was measured by mechanical sieving and classified according to the International Society of Soil Science System (Black, 1968). The following grain size fractions were determined: coarse sand (2–0.2 mm), fine sand (0.2–0.02 mm), silt (0.02–0.002 mm), and clay (<0.002 mm). The study of soils was carried out using standardized techniques of soil prospecting and sampling (F.A.O., 2006; Soil Survey Staff, 2017).

### 2.5 Germination assays

Seeds from 30 plants of the Margalides population were collected near the top of the island, approximately 40 meters above sea level. Donor plants were separated by more than 2 meters from each other. Seeds were collected from the second and third branching levels of the inflorescence, as preliminary analysis had demonstrated that there were no significant differences in germination between these levels. The seeds were mixed and kept in paper envelopes in the dark at room temperature (approximately 20°C, with a relative humidity of 50%–70%). Seeds were disinfected for 10 minutes with a 50% (v/v) solution of bleach (6.25% NaClO) and rinsed 10 times for 1 to 2 minutes with sterile distilled water (Sauer and Burroughs, 1986).

Seed germination experiments aimed to examine the impact of various natural factors that the seeds may encounter in their natural habitat. These included determining the ideal temperature range for germination, observing temporal changes in seed dormancy, exploring methods for breaking dormancy using germination stimulants such as gibberellic acid and potassium nitrate, assessing salt tolerance and examining the effect of osmopriming. Additionally, acid scarification was performed to investigate the possibility of seed dispersal by birds.

In all treatments, a batch of 100 seeds was used; these seeds were distributed into four replicates, each of 25 seeds. These replicates were placed in Petri dishes containing 0.9 ml of sterile distilled water or treatment solution and lined with one Whatman No. 1 filter paper and wrapped in two layers of aluminum foil. Seeds were checked every five days with a low-intensity green light (Baskin and Baskin, 1998). Seeds with a radicle that had emerged more than one seed width (approximately 3 mm) were scored as germinated. On each inspection day, the arrangement of plates containing the seeds was altered, and they were randomly placed in the germination chamber (Hurlbert, 1984). The trials lasted 40 days, after which time the dishes were checked for an additional three weeks to confirm that no more germination had occurred (Baskin and Baskin, 1998). A tetrazolium test was performed on non-germinated seeds to check their viability (Grabe, 1970). Essays on light/darkness, sulfuric acid scarification, thermal shock, caruncle elimination, immersion in marine water, and stress tolerance were carried out with 21 months old seeds.

### 2.6 Temporal variation in seed germination

To determine if *E. margalidiana* seeds are dormant, a batch of seeds was sown in darkness immediately after harvesting. Other batches of seeds kept in paper envelopes under laboratory conditions were sown every 3 months during 2 years of dry storage.

### 2.7 Influence of light on seed germination

To determine whether the seeds are capable of germinating in light, a batch was placed on Petri dishes in an illuminated germination chamber. The assays are 13/11 hours dark/light at 20°C and 11/13 hours dark/light at 10/20°C (light coincides with maximum temperature). The light of the diurnal period was provided by cool white (Osram, Munich, Germany) and Gro-Lux (Sylvania, Erlangen, Germany) fluorescent lights in a 3:2 ratio (light intensity 60 µmol m-2 s-1) (Choy et al., 2008).

### 2.8 Seed germination with sulfuric acid scarification

To simulate the passage of fresh and one-year-old seeds through the digestive tract of animals, a batch of seeds was immersed in concentrated sulphuric acid (96%) for five minutes, then washed 10 times for 1 to 2 minutes with abundant sterile distilled water, and immediately sown (Narbona et al., 2006).

### 2.9 Germination with hormonal treatments using gibberellic acid

Seed germination is a process regulated by hormonal action, and gibberellin can be used to eliminate or reduce the dormancy of species. In a previous germination test, *E. margalidiana* showed no germination with a 100 mM GA_3_ concentration solution. Therefore, germination in response to 0, 0.1, 1, and 10 mM GA_3_ was evaluated. GA_3_ (10 mM) was dissolved in hot water while vortex mixing, and then cooled and filter sterilized before being serial diluted to prepare lower concentrations. Germination was conducted using fresh seeds and standard conditions.

### 2.10 Germination with the application of nitrified solutions

In several species, the stimulatory effect of KNO_3_ application on germination is known (see, e.g., Fawcett and Slife, 1978; Hilton, 1984; Hilhorst and Karssen, 2000 and citations). Due to the ornitocoprophilous origin of the islet substrate, nitrates are abundant in the soil (up to 0.56% N, see soil determinations). To determine a possible effect on the germination of fresh seeds, trials were conducted with KNO_3_ solutions at concentrations of 1 mM, 10 mM, and 100 mM. For this purpose, KNO_3_ (100 mM) was dissolved in water, adjusted to pH 3, and filter sterilized. Lower concentrations were then prepared by serial dilution, maintaining the pH.

### 2.11 Effect of Thermal Treatments on seed germination

To determine the optimal temperature range for germination of *E. margalidiana* we performed germination assays with temperatures from 16°C to 26°C in 12-month-old seeds which have a moderate degree of dormancy. Several thermal treatments were designed to simulate the temperatures reached in the Mediterranean area at ground level in rocky crevices in the summer (Valbuena and Vera, 2002; Navarro and Guitián, 2003; Cortés and Llorens pers. Obs.). For this purpose, batches of seeds were subjected to temperatures of 45°C for 2 hours repeated on 5 successive days, 45°C for 6 hours repeated on 10 successive days, and alternating temperatures of 20:45°C for ten days repeated 3 times. The seeds were sown immediately after the treatments were finished, with three replications per thermal treatment (Cristaudo et al., 2019). Although no known fires have occurred in the islet, treatments aimed at simulating the conditions that occur in the soil surface after fires were designed according to (Trabaud, 1979; Bradstock and Auld, 1995; Narbona et al., 2007). Before sowing, four batches of seeds were placed in an oven, two were subjected to temperatures of 100°C for 5 and 15 minutes, and the other two at 140°C for 5 and 15 minutes, respectively.

### 2.12 Germination after caruncle removal

To simulate the impact of ant jaws on germination, a scalpel was used to remove the caruncle from a batch of seeds before sowing, to study its effect on germination (Pacini, 1990).

### 2.13 Salinity tolerance

A seed lot of 21 months old and another seed lot of fresh seeds were used to examine the ability of seeds to tolerate salinity. Seeds were moistened with the following test solutions: 100%, 50%, 25%, and 12.5% seawater. Seawater was taken from the study site, with a salinity of 3.7 PSU. Seeds germinated in distilled water were used as controls. After 20 days, seeds that failed to germinate in the seawater solutions were transferred to new dishes with fresh water only, to test for germination recovery. Germination was scored for an additional 20 days.

### 2.14 Effect of Osmotic shock on seed germination

To test for osmotic stress tolerance, 21-month-old seed lots were used. Seeds were placed on filter dishes imbibed with 5 ml of osmotic solutions of -0.14, -0.3 and -0.6 MPa, prepared by dissolving appropriate quantities of polyethylene glycol (PEG 6000) in distilled water (Hardegree and Emmkrich, 1990; Balestri and Cinelli, 2004). These osmotic potentials are within the range of those known to affect the germination of several Mediterranean perennial coastal plants (Thanos et al., 1989). Seeds germinated in distilled water served as controls. To test for germination recovery, seeds that failed to germinate in PEG solutions, after 20 days, were transferred to new dishes with fresh water. Germination was scored for an additional 20 days.

### 2.15 Seed dispersion assays

To assess the dispersal capacity of seeds in the sea, we used the method described in Sawada and Tsuda (2005) and Sánchez-Salas et al. (2012) with some modifications. Briefly, two lots of five batches of 25 seeds were prepared, one from freshly harvested seeds and the other from 21-month-old seeds. Each was placed in a container containing seawater for 100 hours. The floating and sunken seeds were then collected separately and immediately sown. Additionally, two batches of 25 unopened capsules collected in the vicinity of the plants were treated in the same way for seven days and their buoyancy was determined. The density of the collected seeds was also determined.

### 2.16 Vegetation analysis

We used 45 floristic inventories on minor islets (less than 8 Ha surface) of Western Mediterranean Sea (Columbrets, Cabrera, and Pityusic and Majorca archipelagos) from a previous study. Vegetation inventories were made according to Braun-Blanquet method (Braun-Blanquet, 1964), with presence and abundance species values ranging from 0.2, for sporadic or rare occurrence, to 5, for coverage of between 75 to 100% of the territory.

### 2.17 Drought tolerance of plants from seeds and vegetatively propagated from stem cuttings

#### 2.17.1 Resistance of the reproductive forms of *E. margalidiana*

The essay was designed to assess the survival under drought conditions of plants derived from seeds and stem cuttings. It was designed to obtain information on the survival capacity in different conditions of individuals of *E. margalidiana* obtained from seeds (developing caudex) and cuttings (not developing caudex). This can provide information on the most valuable propagation strategies to be used in species recovery efforts.

Plants were obtained from seeds collected from the population of plants in Ses Margalides. Stem-cuttings for rooting were prepared from apical segments of cultivated individuals grown on the UIB campus following Luna et al. (2008) in 6.5 L containers (23.5 x 20.5 x 21.5 cm). Two batches of 180 containers were prepared. In one of them, seedlings were planted, and in the other, the rooted cuttings. The sowings were carried out in April and were maintained in optimal conditions for 6 months (November). Then batch was divided into 9 groups of 20 units (9 x 20 seedlings and 9 x 20 cuttings containers). To simulate the effect of summer drought on the native habitat, irrigation was eliminated at three levels. Three seedlings (3 x 20) and three cuttings groups (3 x 20) were watered twice a month for three months; another equal number of plants was watered once for three months, and a third group remained unwatered for three months. After this period (September), regular watering was resumed. Finally, one month after (October), the number of plants that survived was counted in each of the treatments.

#### 2.17.2 Drought tolerance comparison among closely related Euphorbia species

*E. margalidiana*, *E. squamigera*, and *E. dendroides* L. were used for the experiment. The first two species develop caudices of different sizes, while the third one lacked this structure which is commonly associated with a function in the storage of water and nutrients. For each species, three batches of fifteen containers (23.5 x 20.5 x 21.5 cm) were arranged with a two-year-old individual in each of them and were maintained without watering for six months (from April 15th to September 15th). After this period, watering was resumed, and the number of surviving plants was determined one month later.

### 2.18 Volatile analysis of pseudanthia (cyathia)

Scent was sampled from plants of *E. margalidiana* and *E. squamigera* from natural populations and cultivated plants. To compare the flower scent of species that are taxonomically related or occupy similar rupestrian habitats, *E. squamigera* var. *montgoi* O.Bolòs & Vigo (Montgó-Denia, Eastern Iberian Peninsula), *E. papillaris* (Marettimo and Scopello, Sicily), *E. bivonae* (Cefalu and Palermo, Sicily) and *E. dendroides* L. (Mallorca) were additionally sampled. VOCs were collected from 20 plants of *E. margalidiana* and *E. papillaris* and 10 plants for the rest of the taxa.

Floral scent volatiles emitted by flowers (cyathia), were collected using dynamic headspace trapping on Tenax TA (DHS-Tenax TA) method, according to Tomàs et al. (2022). For each determination separately two inflorescences (cyathia) were used. Inflorescences were placed in polyacetate bags sealed and left to equilibrate for 1 h. Volatile compounds from each bag were extracted by pumping the air through a small cartridge filled with 100 mg of Tenax TA at a flow rate of 200 mL/min for a duration of 120 min. An ambient control sample was taken from an empty polyacetate bag sampled for the same duration. Sampling hours ranged from 10 a.m. to 2 p.m., the period in which maximum pollinator visits occur.

TD-GC-MS analysis was performed according to the methodology of Tomàs et al. (2022). An Agilent 6980 GC-MDS 5975 inert XL (Agilent Technologies, USA), equipped with a Supelcowax 10 gas capillary column (60m x 0.25 mm x 0.25 µm) at 1.2 mL/min and an automated thermal desorption system (TD-100, Markes Intern.) was used. The GC oven temperature program was from 50°C to 250°C at a rate of 5°C min^-1^. Helium was used as the carrier gas (1.2 mL min^−1^), and the injector and detector temperatures were set at 250°C. The mass spectrometry (MS) conditions were ion source temperature 200°C, and ionization voltage 70 eV impact mode. The mass scan range was 40–500 amu. MS spectra were obtained using the total ion scan (TIC) mode mass range m/z 45–500 amu. The chromatograms and spectra of the samples were processed using the GC-EM software Turbomass version 5.1 (Perkin-Elmer, Inc.). Scent samples analysis was used to delimit the compounds emitted from flowers and determine the contribution of single compounds to the total scent (percentage amount).

The main volatile compounds were annotated by comparing the mass spectra with mass spectral libraries (Wiley 7th edition and NIST08), and by comparing calculated retention indices with those given by NIST08, Adams (2007), and El-Sayed (2017). The annotation of the main compounds was verified using an in-house developed mass spectra/RI library. The individual peaks of extract components were identified by comparing their Kovats retention indexes relative to C7-C30 saturated alkanes (Kováts, 1958; Van Den Dool and Kratz, 1963; Adams, 2007), computer matching with the NIST MS Search 2.0 library, and comparison of their mass spectra to those of previously published data, literature data, and to a stored laboratory mass spectral library. The major compounds detected in the floral headspace were verified by comparison to authentic samples of the compounds.

### 2.19 Pollinator visits

To determine the pollinators and how often they visited *E. margalidiana* flowers, a census was conducted by observing three plants every hour during daytime for 10 minutes each time in the natural population of Ses Margalides during the intense flowering period. The same approach was used in plants cultivated populations in Santa Agnés (NE of Ibiza Island; 39° 02’20’’N; 1°20’00’’E), which is about 1.8 km from the islet of Ses Margalides. All the insects identified as pollinators were found to have *E. margalidiana* pollen on their bodies.

### 2.20 Statistical analysis

Statistical analysis for germination experiments was carried out using the statistical package Statgraphics Centurion Version 18.1.13. Requirements of ANOVA were checked by normality plots and by testing the homogeneity of variance of residual means (Test of Levene). Prior to variance analysis, data were subjected to a logarithmic transformation. Significance of differences among treatments was tested by applying one-way ANOVA (Abdi and Williams, 2010; Rutherford, 2011).

For vegetation analysis of minor islands, data from 45 inventories was transformed following the criteria of Dagnelie (1960). Taxa with a low presence index (<5% of the relevés) were omitted after checking for chorological, phytosociological, or ecological contributions that were not significant. Vegetation grouping was carried out using the TWINSPAN (Two-way indicator species analysis) method.

For the analysis of volatiles emitted from the pseudanthia (cyathia) from different species, we performed a Principal component analysis (PCA) to determine the relationship between samples, compound groups, and principal factors using Multivariate Statistical Package Version 3.0, 1998, Kovac Computing Service. Variance analysis (ANOVA) was conducted to determine the statistically significant differential compounds between taxa (P < 0.05). Before data normality verification by the Shapiro-Wilks test, data were transformed by the equation y = arcsin √x/100, suitable for percentages or proportions data, and submitted to the Lèvene test to check for normality of the data. A hierarchical clustering analysis was performed by PC-ORD (McCune and Mefford, 2016).

## 3 Results

### 3.1 Vegetation analysis

In order to characterize the vegetation of Ses Margalides, we conducted a plant community analysis based on 45 floristic inventories from minor islets, with a surface area of less than 8 Ha, in the Columbrets, Cabrera, Pityusic, and Majorca archipelagos in the Western Mediterranean Sea. Classification of vegetation was performed using the two-way indicator species analysis (TWINSPAN). The results of the vegetation analysis indicated two types of chamaephytic groups. The first group was identified by *Suaeda vera* Forsskal ex J.F. Gmelin, while the second group consisted of various shrub species such as *Malva arborea* (L.) Webb & Berthel., *Medicago citrina* (Font Quer) Greuter, and *E. margalidiana* (**Figure 3**). However, due to the limited and heterogeneous flora of the islets, we were unable to identify any other homogeneous groups of vegetation. We found that *E. margalidiana* is clustered in the group of species that defines a type of coastal nitrophilous chamaephytic vegetation. These results indicate that, from a phytosociological perspective, the community to which *E. margalidiana* belongs is the alliance Medicagini citrinae-Lavaterion arboreae O. Bolòs, Folch & Vigo in O. Bolòs & Vigo 1984 (Pegano-Salsoletea Br.-Bl. & O. Bolòs, 1958).

### 3.2 Seed germination and dispersal

To evaluate if seeds may be able to survive the fluctuating resource availability in the islet of Ses Margalides, typical of Mediterranean regions, we analyzed the germination capacity of seeds of different ages. The analysis of the evolution of dormancy (**Table 2**) shows a significant decrease in the dormancy rates from month 6 onward, reaching its minimum value in month 21.

**Table 2:** Germination rates of seeds of different ages of *E. margalidiana* collected from the natural population of Ses Margalides.

Months	Mean ± s.d.
0	5.3 ± 1,8
3	5.0 ± 1.6
6	6.2 ± 1.1
9	35.7 ± 3.9
12	64.5 ± 5.9
15	69.8 ± 2.9
18	75.3 ± 3.4
21	94.8 ± 2.0
24	90.2 ± 1.2

Four replicates of 25 seeds were used for each assay. Assays were performed at 20°C. The values in the table represent the mean germination percentage along with the corresponding standard deviation.

We next aimed to determine the optimal temperature for germination. Germination tests carried out with 12 months old seeds revealed that the maximum germination rates are reached at temperatures between 18-22°C (**Table 3**) with no significant differences in seed germination between light or dark conditions at optimal germination temperatures (**Table 4**; T_50_ values: **Supplemental Table 1**). We next investigated the mechanisms of dormancy using gibberellic acid. We found that GA_3_ treatments with fresh seeds influenced germination, with GA_3_ at 10 mM concentration being particularly effective in promoting it (76.1% versus 5.1% of the control). Similarly, the treatment of seeds with KNO_3_ showed an effect on dormancy. A 10 mM KNO_3_ treatment increased the germination rate of dormant seeds from 5.1% to 28.8%. Altogether these results indicate that fresh seeds are mature but the presence of endogenous inhibitors prevents their germination.

**Table 3:** Optimal germination temperature.

Temperature (°C)	Germination (%)	T_50_ (days)
16	30.9 ± 4.59	8.5 ± 0.44
18	57.7 ± 3.42	6.7 ± 0.37
20	62.4 ± 5.60	5.1 ± 0.11
22	59.2 ± 3.16	4.8 ± 0.22
24	26.8 ± 0.71	4.1 ± 0.24
26	0	–

Germination percentage of 12-months-old seeds across a range of temperatures between 16°C to 26°C and T50 values (number of days taken for 50% of total germination).

**Table 4:** Germination rates of fresh and 21-month-old *E. margalidiana* seeds with different treatments.

	Fresh seeds	Seeds 21 months old	
Treatment	Germination (%)	Germination (%)	Recovery
Control	5.1 ± 0.2	96.2 ± 2.8	–
Sulphuric acid scarification	0.9 ± 0.5	3.9 ± 2.6*	–
100°C x 5 min	2.3 ± 1.1	74.8 ± 4.4	–
100°C x 15 min	1.0 ± 0.9	48.8 ± 6.9*	–
140°C x 1 min	1.0 ± 0.8	21.6 ± 1.3*	–
140°C x 15 min	0	7.5 ± 4.4*	–
GA3 0.1mM	4.3 ± 0.7	–	–
GA3 1mM	32.4 ± 3.1*	–	–
GA3 10mM	76.1 ± 1.2*	–	–
45°C x 2h x 5 days	21.2 ± 5.9	–	–
45°C x 6h x 10 days	36.8 ± 2.2*	–	–
45°C x 10 days x 3 times	85.3 ± 2.7*	–	–
1 mM KN0_3_	5.1 ± 0.4	–	–
10 mM KN0_3_	28.8 ± 0.6*	–	–
100 mM KN0_3_	18.3 ± 0.8	–	–
Light	–	94.6 ± 4.3	–
Floating seeds	–	0	–
Caruncule removal	–	97.2 ± 4.7	–
12,5% sea water	–	22.6 ± 7.2*	57.7 ± 2.6
25% sea water	–	2.5 ± 2.3*	55.3 ± 6.8
50% sea water	–	0	26.6 ± 3.6
100% sea water	–	0	22.3 ± 3.7
-0.14 MAp	–	91.1 ± 3.1	65.3 ± 4.1
-0.3 MAp	–	10.0 ± 1.2*	68.8 ± 2.4
-0.6 Map	–	0	84.6 ± 1.9

Asterisks indicate significant differences compared to the respective control group with a significance level of P < 0.05.

We next examine the effect of high temperatures that occur in the Ses Margalides. Repeated treatments at 45°C and, especially those of alternating temperatures (20°C/45°C) promote germination with the most effective treatment reaching germination values of 85.3% (**Table 4**). Although no fires have been reported on the islet of Ses Margalides, we decided to further investigate the potential impact of thermal shock on seed dormancy. To test this, we subjected seeds to a brief thermal shock treatment ranging from 100-140°C. However, we found that this treatment had no significant effect on seed dormancy (**Table 4**). We further investigated seed germination under saline conditions and found that salt concentrations have a significant impact on germination. When seawater was used in a dilution of 12.5%, we observed that slightly over 20% of the seeds were able to germinate but this value rapidly declined in treatments with increasing seawater concentrations (**Table 4**). These findings suggest that the ability of seeds to germinate under saline conditions is limited, with moderate levels of salt having a negative effect on germination rates. Additionally, we investigated if osmopriming with PEG could improve seed germination. All tests showed a decrease in germination which was strongly inhibited at -0.3 Mpa concentration and completely abolished at -0.6 Mpa (**Table 4**).

We next investigated seed dispersion mechanisms which determine the spatial distribution of the species and its ability to colonize new areas outside the islet. We first investigated dispersion by seawater and found that neither fresh nor 21-month-old, well-developed seeds (ρ = 1.253 ± 0.042) float (buoyancy 0.03 ± 0.06) in seawater (ρ = 1.027). However unopened seed capsules containing 1-3 seeds, which could eventually detach from the plant and fall to the sea before opening, can float for a short period (3.1 ± 1.2 days, n = 50) before sinking.

To evaluate the possibility of endozoocory we performed acid scarification assays. The results show that there is no increase in the germination of dormant fresh seeds. Additionally, in 21-month-old seeds, this treatment caused a strong decrease in germination. Furthermore, tetrazolium testing (TZ) conducted on treated seeds revealed that non-germinated seeds had lost germination power (**Table 4**).

Finally, we aimed to assess the impact of ant dispersal on seed germination and the distribution of *E. margalidiana* in the islet. To simulate the removal of the caruncle by ant jaws, a scalpel was used to remove the caruncle from a batch of seeds before sowing. The results showed that the presence or absence of the caruncle did not affect seed germination (**Table 4**). This suggests that ant dispersal may not play a significant role in the distribution and germination of *E. margalidiana* on the islet.

### 3.3 Drought tolerance

**Figure 4:**
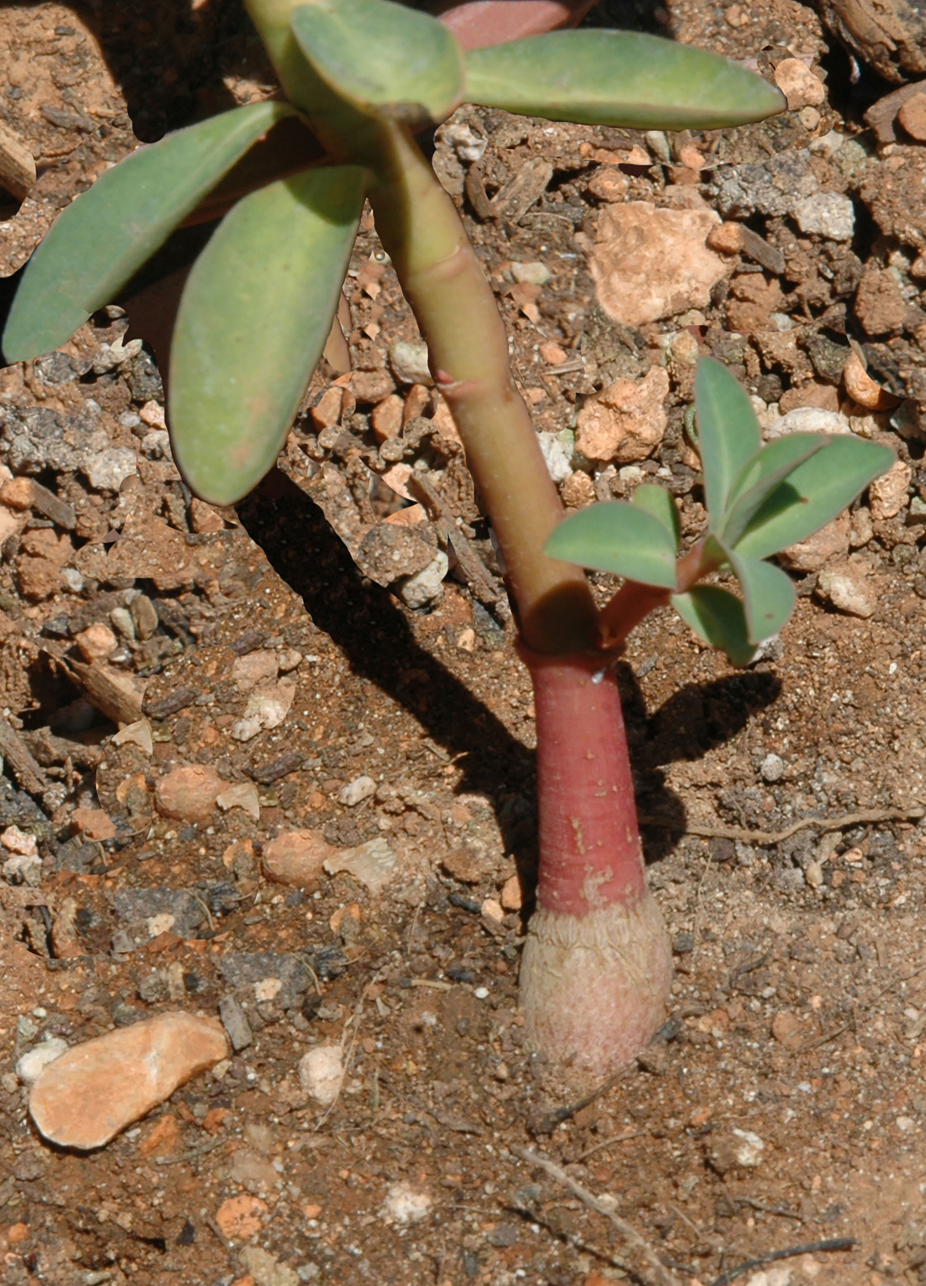
Detail of the hypocotylar caudex of *E. margalidiana*. Photos taken by the authors.

We next investigated the drought tolerance of *E. margalidiana* plants obtained from seeds and those obtained from vegetative propagation. We found that seed-derived plants outperformed those obtained through vegetative propagation indicating that the presence of a hypocotyl caudex is important for survival during drought conditions (**Figure 4**, **Table 5**). In treatments A and B, which represented the closest conditions to those found on the Ses Margalides islet, 94.4% and 70.2% of the seedlings with caudex survived, while only 31.5% and 16.6% of the cuttings survived, respectively. These results highlight the importance of seed-derived plants in adapting to harsh environmental conditions.

**Table 5:** Seedlings vs cuttings drought survival test.

Assay	A (%)	B (%)	C (%)
**Seedlings**	93.4 ± 3.5	70.2 ± 2.1	55.2 ± 3.1
**Cuttings**	31.5 ± 3.8	16.6 ± 2.2	3.0 ± 1.2

Treatment A: 2 watering x month; Treatment B: 1 watering x month; Treatment C: No watering. Average percentage of surviving plants ± s.e (n=60). The duration of the experiment was 3 months.

We further investigated the function of hypocotyl caudex on drought tolerance. We compared three closely related species of Euphorbia, two capable of forming a caudex and the other not, during a drought test. The findings showed that plants with a caudex had higher survival rates, while those without, namely *E. dendroides*, had lower survival rates after severe drought stress (**Table 6**).

**Table 6:** Euphorbia species drought tolerance comparison.

	*E. dendroides*	*E. margalidiana*	*E. squamigera*
Experiment 1 (15 plants)	1	9	4
Experiment 2 (15 plants)	2	9	3
Experiment 3 (15 plants)	2	10	5

Number of plants surviving after no irrigation for 5 months (April to August) in three independent experiments.

### 3.4 Pseudanthium (cyathium) scent composition in closely related Euphorbia species

**Figure 5:**
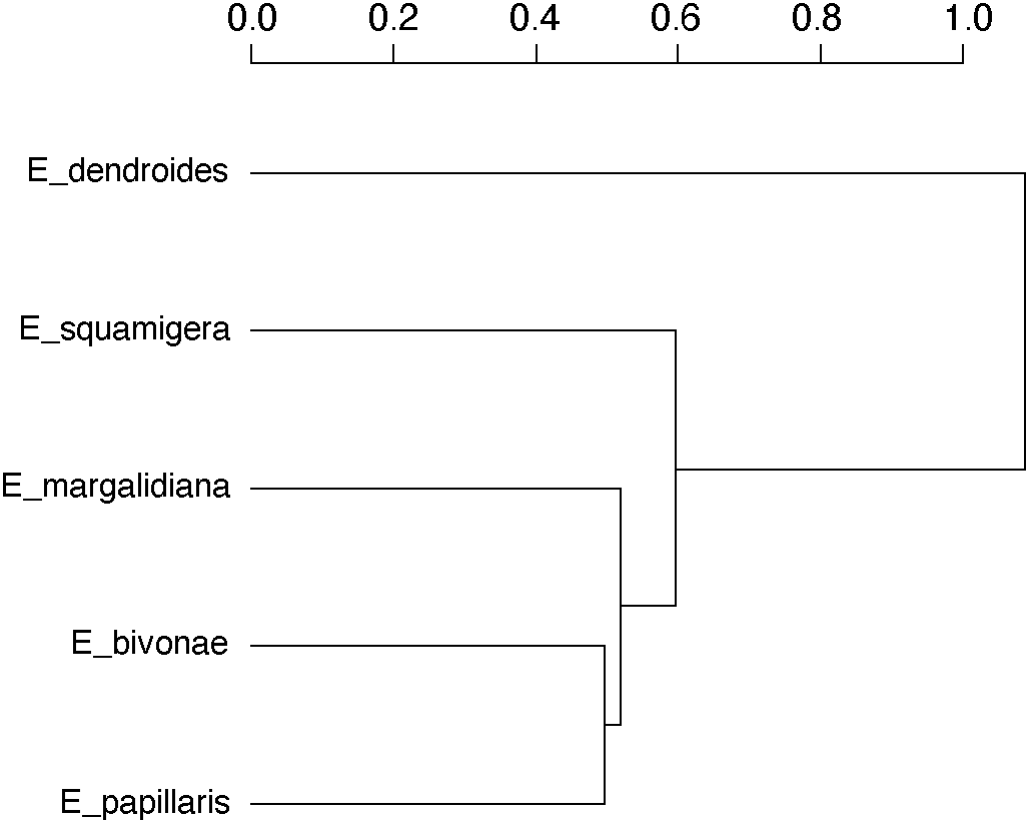
Hierarchical clustering (Jaccard distance measure, average linkage method) of species samples based on floral volatile profiles.

**Figure 6:**
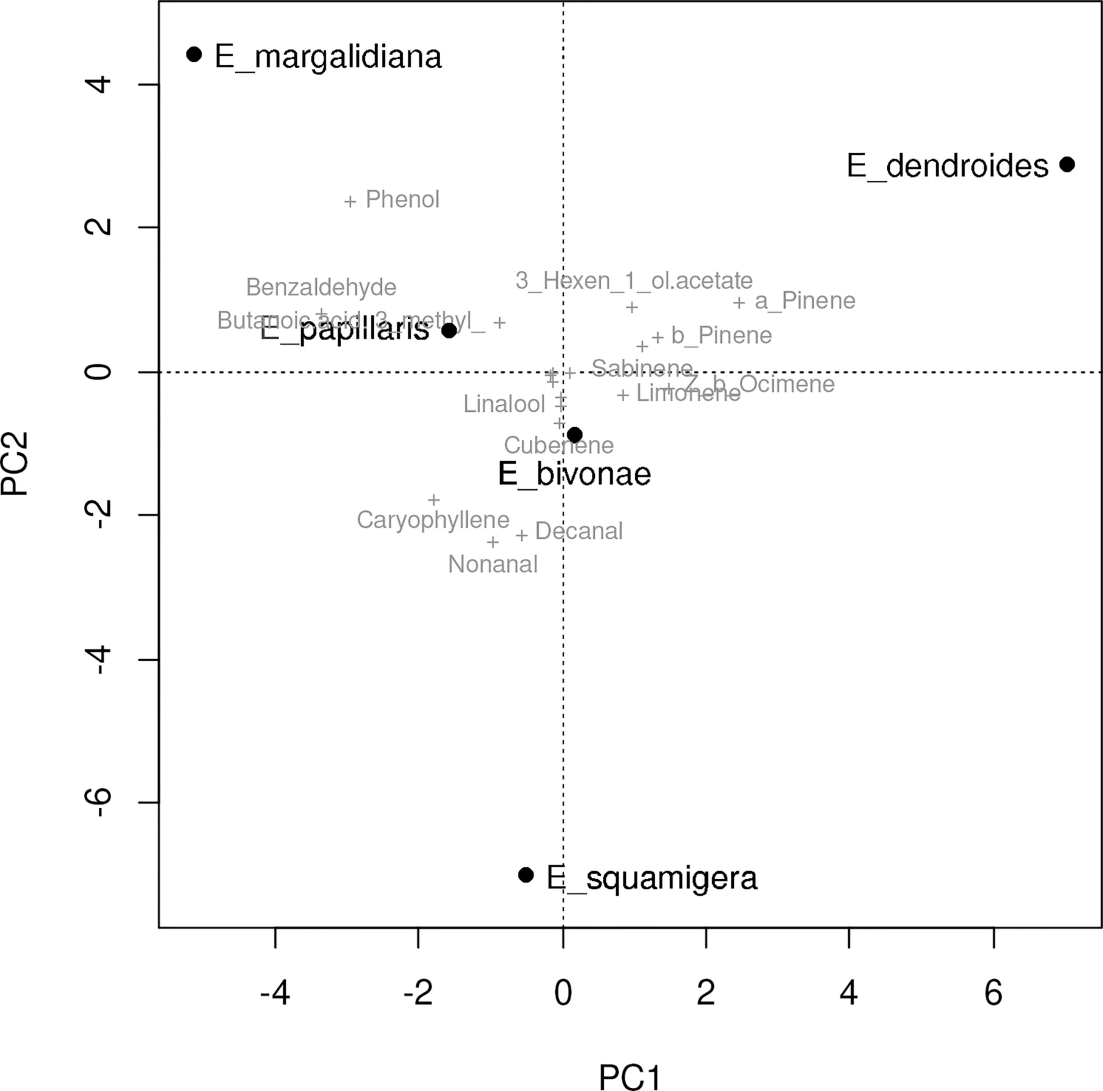
PCA biplot based on the Bray Curtis distance matrix of the mean contribution of floral volatiles for each Euphorbia species.

The volatile profile of four closely related Euphorbia species was analyzed to identify unique chemical signatures. The results revealed significant differences in the volatile profiles of the studied species (**Table 7**). In *E. margalidiana*, in addition to the low presence of monoterpenes, the relative abundance of the sesquiterpene caryophyllene and some aliphatic compounds highlights, as well as the importance of benzene derivatives, among which benzaldehyde and, especially, phenol stand out. This compound characterizes and individualizes this species compared to the others, so that only a regular presence has been determined, and in a lesser proportion in *E. papillaris*, and *E. bivonae*, although in this species it is only found irregularly and in much smaller quantities. On the other hand, *E. dendroides* is the only species in which monoterpenes are predominant (over 85%). Statistical analysis of the samples indicates a clear differentiation between the different species based on floral volatiles (**Figures 5**, **6**), differences driven by some specific compounds. The analysis reveals a clear individualization of *E. dendroides*, which is mainly based on monoterpene abundance. A similar process occurs in *E. margalidiana* and to a lesser extent in *E. papillaris*, with the separation primarily due to the high proportion of benzenoids, particularly phenol and benzaldehyde.

**Table 7:** Cyathia scent of four Euphorbia species subsect.

Compounds /Species	*E. margalidiana*			*E. squamigera*			*E. bivonae*			*E. papillaris*			*E. dendroides*		
	M	Max/Min	P	M	Max/Min	P	M	Max/Min	P	M	Max/Min	P	M	Max/Min	P
Monoterpenoids and rel.															
α-Pinene													27.5	35.1/14.1	100
β-Pinene													14.7	15.4/7.6	100
α-Thujene							10.7	15.9/2.3	80	8.2	10.6/5.8	75			
Sabinene							3.4	3.8/0.8	50				12.2	16.7/11.9	100
Limonene	1.6	7.5/1.2	30	8.9	12.7/6.1	40	10.1	20.0/0.6	100	10.2	12.3/5.4	100	13.1	26.3/8.8	100
(Z)-β-Ocimene				8.8	20.0/2.4	40							17.2	20.2/2.0	90
cis-Linaloloxide				3.1	4.9/1.7	60									
Linalool				4.2	6.6/2.6	40	2.3	3.4/1.1	40						
Sesquiterpenoid															
Cubenene				7.4	9.6/5.5	40									
Caryophyllene	24.8	47.2/3.5	45	31.9	42.5/23.0	70	21.4	47.3/0.7	70						
*Aliphatic compounds*	* *														
*Aliphatic acids*															
Butanoic acid, 3-methyl-	13.2	49.1/3.3	55												
*Aliphatic alcohols*															
3-Hexen-1-ol	0.5	5.1/1.9	10										6.3	12.3/3.9	100
1-Hexanol, 2-ethyl	2.9	21.9/7.7	15				11.1	29.0/0.8	60						
Aliphatic aldehydes															
Nonanal	7.7	32.8/1.4	75	30.7	39.8/19.0	70	9.5	15.0/1.9	40	14.4	17.5/11.1	100			
Decanal	2.3	13.3/0.8	35	27.1	42.2/12.3	50				12.7	14.5/8.7	100			
Aliphatic esters															
3-Hexen-1-ol, acetate	7.0	5.5/0.8	20										15.9	22.6/10.3	100
*Benzenoids and rel.*															
Benzaldehyde	38.7	63.8/9.6	90	15.3	60.5/1.1	70	19.4	43.2/3.5	40	37.7	53.0/21.1	100			
Benzylalcohol	0.7	3.3/1.1	25				3.7	4.5/0.6	80				1.5	3.0/0.5	70
Phenol	41.7	52.9/22.9	100				1.7	2.1/1.4	60	11.4	13.9/7.3	100			

Galarrhaei (Boiss.) Pax and *E. dendroides*. Compounds expressed as a percentage of total presence. VOCs indicated are present in at least two species or in more than 40% of samples from one species. M, mean; Max, maximum; Min, minimum; P, occurrence (%); (n=20) for *E. margalidiana* and *E. papillaris*; (n=10) for *E. squamigera*, *E. bivonae* and *E. dendroides*.

### 3.5 Pollinators

**Figure 7:**
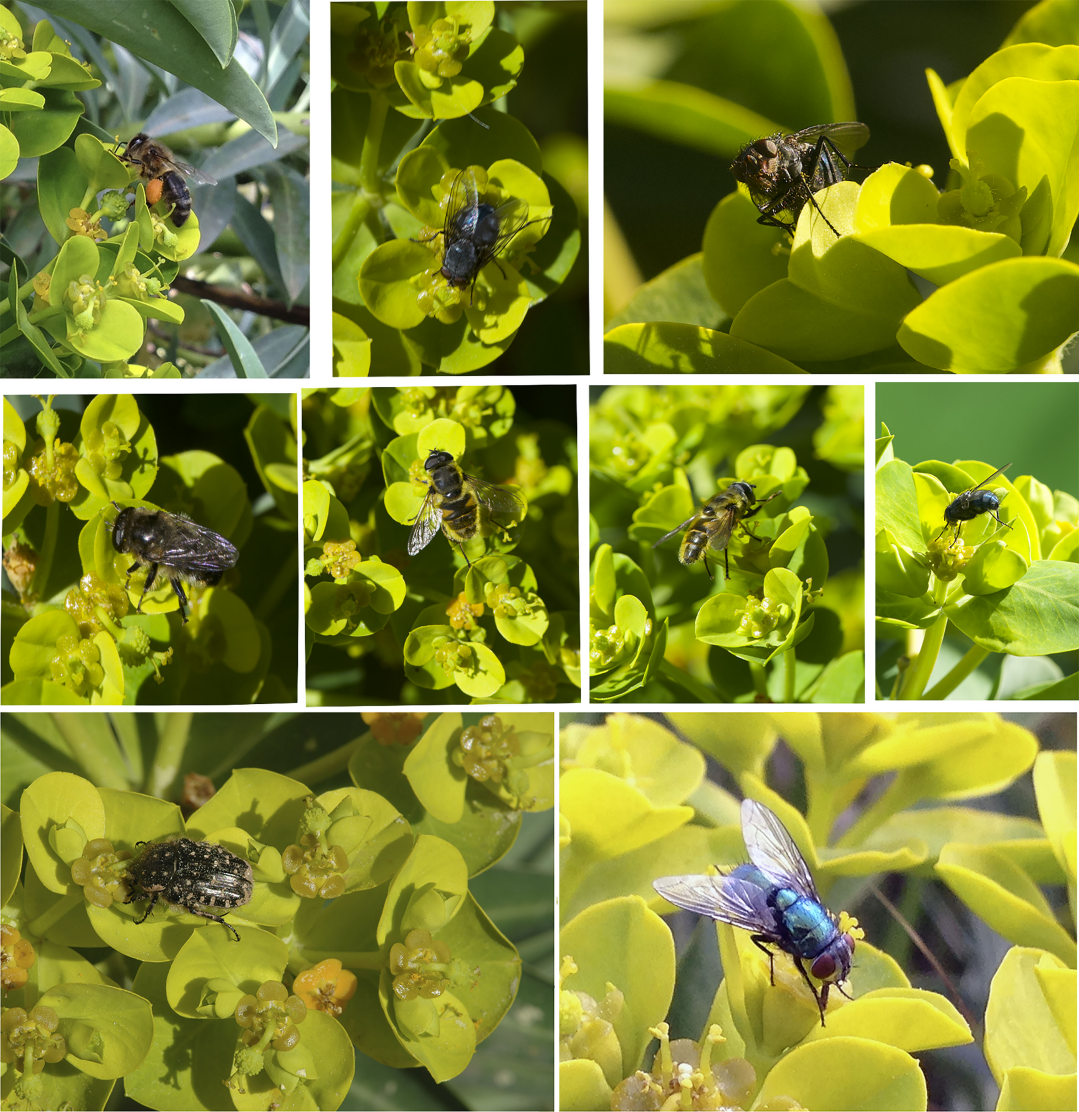
Pollinator diversity in *E. margalidiana*. Photos taken by the authors.

We aimed to determine the main pollinators of *E. margalidiana* by conducting observations of the wild population on Ses Margalides and cultivated plants in the nearby area of Ibiza (**Table 8**). These observations allowed us to gain a broader understanding of the pollination potential of this species, which could have been missed if we only observed the natural population due to the lack of other potential pollinators. Our findings showed that flies were the main and almost exclusive pollinators on the islet, while bees and other insects were observed as pollinators in cultivated plants (**Figure 7**).

**Table 8:** Main pollinators groups of *E. margalidiana* in Ses Margalides and in cultivated plants (near coastal Ibiza, Santa Agnés; 39° 02’20’’N; 1°20’00’’E).

Pollinator group/Sample	1	2	3	Total	%
Margalides					
Diptera (Calliphoridae)	60	41	40	141	99.3
Other Diptera (Syrphidae)	0	1	0	0	0.7
Apinae	0	0	0	0	0
Others	0	0	0	0	0
Cultivated					
Diptera (Calliphoridae, Muscidae)	124	62	79	101	43.9
Other Diptera	22	18	21	81	17
Apinae	37	33	43	113	27.1
Others	17	16	17	50	12

## 4 Discussion

### 4.1 Microclimatic and edaphic conditions of Ses Margalides and its relation with the vegetation of other islets

Mediterranean islands are recognized as territories with a key role as biological refuges and enclaves (Médail, 2016). In them, the effects of isolation and peculiar ecological conditions on the evolution of plants are also very evident (Fernández-Palacios et al., 2021). The islets, conditioned by the influences derived from their proximity to the sea, are spaces where only a very limited number of habitats exist, each one determined by ecological conditions that cause strong stress effects on plants, resulting in low diversity of species (Bellard et al., 2012). Furthermore, the variable and irregular incidence of ecological agents determines that the floristic composition of its vegetation is not stable. This is exemplified by therophytic plant communities as they are more predominant as the stability of the habitat increases (**Figure 3**).

In the islets, the intense activity of seabirds on the surface of the colonies determines the morphological and chemical properties of the substrates and are distinct from the climatic context (García et al., 2007). These changes determine the development of various types of vegetation, such as that in which *S. vera* predominates (Tébar et al., 1989; García et al., 2002). However, in Ses Margalides, as in other Mediterranean islets, which have a strongly rupicolous and stressful character, although relatively stable, only halophytic or nitrophilic shrub vegetation can develop (García et al., 2002). This is found only in the summit areas (**Supplemental Figure 1**), where the substrate accumulates in the rocky crevices. The soils in these limestone fissures are sandy-loam with high contents of organic matter. They originate from plant debris, seabird nesting residues, and feces (guano), as well as the mineral particles that they can retain. In the Western Mediterranean, this nitrophilic vegetation is characterized by the development of different perennial or perennating species. Some of them have a wide distribution, such as *Malva arborea* (W. Europe, Mediterranean Region and Macaronesian Region), but others are microinsular endemisms, such as *Diplotaxis ibicensis* (Pau) Gómez-Campo, *E. margalidiana* (only found in Margalides) and *Medicago citrina*. Of these, only *E. margalidiana* has succulent stems and a hypocotyl caudex similar to *Jatropha* (Romero, 2022), which are structures related to xeric and warm places (Hearn et al., 2013; Evans et al., 2014) and which enable plants to sprout and regrow (Ornduff, 1969) as they serve to store water and nutrients.

### 4.2 Seed germination and dispersion

*E. margalidiana* shows non-deep physiological dormancy so that the germination of most seeds begins after a maturation period (12 months to reach 50% germination, and 21 months to reach the maximum level). The existence of dormancy in margalides spurge seeds is evidenced by the potassium nitrate and gibberellic acid treatments, which increased the germination percentage compared to the controls. These effects with potassium nitrate and gibberellic acid are well known in other species (Giba et al., 1995), which are characterized by certain physiological dormancy mechanisms. Nitrate regulation induces nitrate-dependent gene expression, which triggers a nitrate-induced abscisic acid decrease that permits seed germination (Duermeyer et al., 2018). In addition, gibberellic acid is known to decrease the dormancy associated with the immature embryo (McDonald, 2002).

The optimal temperature range for germination is between 18 and 22°C. Below or above these values, germination levels decrease, and at 26°C germination is thermally inhibited. Furthermore, light does not appear to significantly influence germination. These common germination characteristics seem to be quite similar with those of *E. bivonae* (Cristaudo et al., 2019) and with some other Mediterranean species, “Mediterranean germination syndrome” (Skordilis and Thanos, 1995), although in a thermophilic trend group. The adaptability of the germination period (temperatures and dormancy) determines that in a Mediterranean climate such as that of the Ses Margalides islet, germination should mainly occur during the autumn-early winter period, associated with the initiation of the post-summer rainy season. During unfavorable periods (deep winter and summer), germination is blocked either by thermo-inhibition or by thermo-dormancy. This estimation agrees with the germination results in which the seeds of *E. margalidiana* show very low or no germination when water potentials are equal to or less than -0.14 MPa. If this is associated with the natural climatic conditions of the islet, the sporadic rains that occur in late summer, before the start of the rainy season, could act as a pre-treatment that would favor rapid germination afterward. Other peculiarities of the germination of *E. margalidiana* determine the dispersal of the diaspores and the exclusive presence of this spurge on the islet. Thus, the important loss of the capacity of germination of the seeds with solutions of seawater, which agrees with the non-halophilic character (Cardona, 2017), the non-floatability of the seeds and the scarce capacity of permanence on the surface of the water of the fruits that do not open (which rarely occur), determine that practically the thalassochory can be discarded as a viable dispersal mechanism for this species. As a consequence of the high decrease in germination capacity of the scarified seeds with sulfuric acid, a negative estimation is determined concerning the possibility of endozoocory. In contrast, the decrease of dormancy that occurs in the treatments with KNO_3_, agrees with the characteristics of the nitrophilic habitat in which the species prospers.

### 4.3 Comparative drought tolerance of seedlings and cuttings

Survival tests with seedlings and cuttings confirm that plants obtained from seeds show higher drought tolerance than cuttings. This fact, as well as the differences in survival values between *E. margalidiana*, *E. squamigera*, and *E. dendroides* could be interpreted as a consequence of having a hypogeal caudex, which is especially relevant in *E. margalidiana* (**Figure 7**). This structure, which has been shown to promote the survival of other succulent species, is also likely to contribute to the adaptation of *E. margalidiana* to the extreme conditions of the islet. As a result, seedlings and seeds should be used as preferred materials in population reinforcement or propagation programs in xeric habitats, in particular under the climate change scenario, which is expected to cause progressively drier and warmer conditions.

### 4.4 Flower scent and pollinators

As the entomofauna on the islet is scarce, the variation of insects with the potential to act as pollinators is very limited. However, the nesting of seabirds (mainly *Larus michaelis*), sometimes with a significant amount of dead animal remains, supports an abundant population of flies, mainly *Lucilia sericata* (Diptera, Calliphoridae).

Entomophilous plants use signals to attract pollinators. The most common are olfactory and visual, but there are also other types, such as tactile, gustatory, thermal, electrostatic, or acoustic (Von Helversen and Von Helversen, 1999; Balamurali et al., 2015). Based on the signal, the pollinator determines the probability of visiting the plant (Sun et al., 2018). In *E. margalidiana*, the adaptation of the floral aroma of the pollinators constitutes one of its most peculiar features. In the aroma of *E. margalidiana* (**Table 7**), the dominance of only a few components is notable, in particular benzenoids, with benzaldehyde being the major compound (between 63.8-9.6%) and phenol (between 52.9-22.9%), as well as aliphatic compounds, mainly butanoic acid, 3-methyl (49.1/3.3), nonanal, and decanal (32.8/1.4 and 13.3/0.8, respectively), and the scarce presence of monoterpenes (only limonene). This profile presents a remarkable similarity to that of various sapromyiophilous species, such as diverse (Johnson et al., 2020). In particular, the abundance of phenol, only known in similar proportions in *Duvalia corderoyi* N.E.Br. (48.3% phenol) and in lesser proportions in other plant species, sapromyiophilous and non-sapromyiophilous, such as *Orbea verrucosa* (Masson) L.C. Leach and *Euphorbia grandicornis* Goebel ex N.E.Br., respectively (Jürgens et al., 2006; Johnson and Jürgens, 2010). The similarity of the basic chemical profile of the latter species (Aliphatic aldehydes 36.3%: nonanal 15.8%, decanal 20.5%; Benzenoids 43.3%: benzyl alcohol 5.9%, phenol 5.5%; Monoterpenoids: β-linalool 1.4%; Nitrogen-containing compounds: 2-phenylacetonitrile 19.0%) to that of *E. margalidiana* is high, differing mainly in the content of nitrogen-containing compounds (19% in *E. grandicornis* and absent in *E. margalidiana*) (Johnson and Jürgens, 2010).

Phenol is a compound that can be found naturally in the degradation products of plants and animals, such as litter and feces (Li et al., 2016). Moreover, mixtures of phenol and related compounds are known to attract different fly species, such as *Stomoxys calcitrans* (Tangtrakulwanich et al., 2015; Zhu et al., 2016), Drosophila (Den Otter and van der Goes van Naters, 1993), and also screw-worm substrates containing phenol, p-cresol, and indole attract the *Lucilia sericata* fly (Chaudhury et al., 2015). In addition, by mimicking the odor of feces, releasing phenol, cresol, and indole, plants use this attraction to attract flies, which use the feces as a food source or breeding place, exploiting these insects as pollinators by developing sapromiophilous flowers.

The main pollinator of *E. margalidiana* is *Lucilia sericata*. Decaying matter (fruit, meat, garbage dumps, feces, and dead animals) in moist, loose soils are preferred sites for this fly and are coupled to its life cycle, from the larval phase to adults with necrophagous habits (Charabidze et al., 2013; Hauze, 2020).

The potential pollinating entomofauna attracted to the flowers of *E. margalidiana* is wide and diverse. This is exemplified by the visits to the flowers of cultivated plants outside the islet which include 44% of flies (Calliphoridae and Muscidae) while those are practically restricted to the sapro-coprophagous *Lucilia sericea* (**Table 8**) in Margalides. This would support the hypothesis that the attractants of this plant act more on the perceptual biases of the visitors, the saprophilous flies associated with nesting seabirds, than on mimicry. Further antenogram and behavioral response analysis should elucidate this question. Nevertheless, the response of this species denotes a unique and appropriate adaptation to the conditions of the islet.

The survival and endemicity of *E. margalidiana* in the islet of Na Foradada can be attributed to several factors: 1) Succulence and presence of a hypocotyl caudex which allows it to survive under severe drought conditions, 2) Its nitrophilic character which allows outcompeting other plant species; 3) Difficulties or impediment to disperse outside the islet with no seed and poor fruit buoyancy, poor tolerance to germinate in saline conditions (non-haline character, no endozoochorous dispersal); 4) Floral scent adapted to attract flies, possessing a volatile profile similar to that of sapromiophilous flowers, which mimics fecal and decomposition odors of seabird nesting remains. The main compound phenol is an attractant for flies, which are the main and almost exclusive pollinators on the islet. A distinctive chemical trait that highlights the key adaptive processes that have allowed this endangered plant species to thrive in this extreme habitat.

## Data availability statement

The raw data supporting the conclusions of this article will be made available by the authors, without undue reservation.

## Author contributions

All authors contributed to the development and writing of the manuscript. All authors read and approved the final manuscript.
