# Supplementary material for: Deciphering the ecology of the threatened microendemic species Euphorbia margalidiana
Source: Front Plant Sci. 2023 Jun 26;14:1155896. doi: 10.3389/fpls.2023.1155896 (PMC10332272; doi:10.3389/fpls.2023.1155896)

Supplemental Figure 1. Vegetation zoning scheme of the major islet of Ses Margalides (Na Foradada)

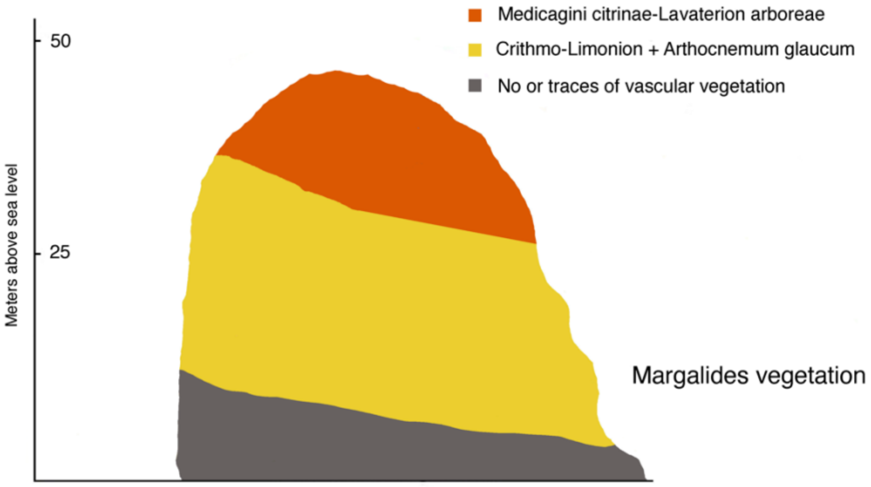

Supplement: Supplementary file 1 [file DataSheet_1.pdf]
