# Supplementary material for: Deciphering the ecology of the threatened microendemic species Euphorbia margalidiana
Source: Front Plant Sci. 2023 Jun 26;14:1155896. doi: 10.3389/fpls.2023.1155896 (PMC10332272; doi:10.3389/fpls.2023.1155896)
Supplement: Supplementary file 2 [file DataSheet_2.pdf]

Supplemental Table 1. T_{50} in days for germination of fresh and 21-month-old *E. margalidiana* seeds with different treatments

	Fresh seeds	Seeds 21 month old
Treatment	t_{50} (days)	t_{50} (days)
Control	7.2 ± 0.3	5.5 ± 0.1
Sulphuric acid scarification	8.1 ± 2.9	8.3 ± 1.7
100ºC x 5 min	8.3 ± 3.2	6.5 ± 0.8
100ºC x 15 min	7.7 ± 4.3	6.2 ± 1.0
140ºC x 1 min	7.6 ± 2.2	6.3 ± 0.6
140ºC x 15 min	0	17.1 ± 4.4
GA3 0.1mM	20.8 ± 1.1	-
GA3 1mM	13.3 ± 2.6	-
GA3 10mM	9.2 ± 0.5	-
45ºC x 2h x 5 days	7.7 ± 1.2	-
45ºC x 6h x 10 days	7.4 ± 2.5	-
45ºC x 10 days x 3 times	5.5 ± 0.7	-
1 mM $KN0_3$	8.9 ± 0.7	-
10 mM $KN0_3$	9.2 ± 0.3	-
100 mM $KN0_3$	12.3 ± 0.5	-
Light	-	5.4 ± 1.5
Floating seeds	-	0
Caruncule removal	-	5.3 ± 0.3
12,5% sea water	-	9.3 ± 3.7
25% sea water	-	14.2 ± 5.3
50% sea water	-	0
100% sea water	-	0
-0.14 MAp	-	7.2 ± 0.9
-0.3 MAp	-	9.4 ± 1.3
-0.6 Map	-	0
